# Full moonlight-induced circadian clock entrainment in *Coffea arabica*

**DOI:** 10.1186/s12870-020-2238-4

**Published:** 2020-01-15

**Authors:** J-C. Breitler, D. Djerrab, S. Leran, L. Toniutti, C. Guittin, D. Severac, M. Pratlong, A. Dereeper, H. Etienne, B. Bertrand

**Affiliations:** 1 CIRAD, UMR IPME, F-34398 Montpellier, France; 2 UMR IPME, Univ. Montpellier, CIRAD, IRD, F-34394 Montpellier, France; 3 INECOL, Clúster BioMimic, 34394 Xalapa Enríquez, Ver Mexico; 4 CNRS, Montpellier GenomiX, c/o Institut de Génomique Fonctionnelle, 141 rue de la Cardonille, Cedex 34 Montpellier, France

## Abstract

**Background:**

It is now well documented that moonlight affects the life cycle of invertebrates, birds, reptiles, and mammals. The lunisolar tide is also well-known to alter plant growth and development. However, although plants are known to be very photosensitive, few studies have been undertaken to explore the effect of moonlight on plant physiology.

**Results:**

Here for the first time we report a massive transcriptional modification in *Coffea arabica* genes under full moonlight conditions, particularly at full moon zenith and 3 h later. Among the 3387 deregulated genes found in our study, the main core clock genes were affected.

**Conclusions:**

Moonlight also negatively influenced many genes involved in photosynthesis, chlorophyll biosynthesis and chloroplast machinery at the end of the night, suggesting that the full moon has a negative effect on primary photosynthetic machinery at dawn. Moreover, full moonlight promotes the transcription of major rhythmic redox genes and many heat shock proteins, suggesting that moonlight is perceived as stress. We confirmed this huge impact of weak light (less than 6 lx) on the transcription of circadian clock genes in controlled conditions mimicking full moonlight.

## Background

Beyond tales and legends, there is no longer a doubt that solar radiation reflected by the moon can be perceived by many organisms on Earth, and the informational role of moonlight as an environmental cue is not questioned [1]. Moonlight and the lunar cycle can affect reproduction, communication, foraging and predation in invertebrates, birds, reptiles, and mammals [1, 2].

Peter W. Barlow’s work clearly demonstrated the impact of local gravimetric oscillations on plant growth and development. These gravimetric variations, i.e. the lunisolar gravity cycle or lunisolar tide, occur daily as result of the impact of the sun and moon on the earth’s surface gravity. Lunisolar tide influences plant phenomena such as leaf movement, stem elongation, fluctuations in tree stem diameter, root growth, biophoton emission by seedlings, and chlorophyll fluorescence [3]. Recently, Gallep and co-workers demonstrated co-variation between ultra-weak light emission, coffee seedling growth patterns and lunisolar gravity cycles [4]. These authors corroborated results previously found in seedlings of other species [3]. The moon’s influence on plant growth and development is well documented with regard to its action on local gravity, but it could also have an effect through the sunlight it reflects.

Light is crucial for plant life, and perception of the light environment dictates plant growth, morphology, and developmental changes. Although plants are highly photosensitive, very few studies have explored the effect of moonlight on plant physiology, and most of the results have generally been conflicting. Between 1926 and 1935, Kolisko showed that the particular phase of the moon at sowing time influences the period and percentage of germination as well as the subsequent plant growth [5–7]. Charles Darwin studied the nyctinastic movement of leaves during the night and concluded that this phenomenon was caused by radiation from the sky [8]. Thanks to the work of Peter W. Barlow, we now know that in most of these studies the influence of the moon was due to its local effect on gravimetry, not to moonlight. But the hypothesis of an influence of moonlight on plants does not seem as foolish when we consider that coral can perceive blue light from the moon, which in turn induces gametogenesis and spawning [9]. Bünning and Mose in 1969 hypothesized that a light intensity as low as 0.1 lx (equivalent to the light from a very small candle) can influence photoperiodism in plants [10]. They suggested that nyctinastic leaf folding in legumes could be a means of preventing moonlight from activating the red form of the pigment phytochrome in the upper leaf epidermis. Following this pioneering study, several recent studies have highlighted the effects that artificial light can have on plants at night. Artificial lighting (also sometimes referred to as light pollution) alters natural light regimes (spatially, temporally, and spectrally), when light is perceived as an information source and not as a resource [11, 12]. Kadman-Zahavi and Peiper (1987) reported that, in their experimental conditions, plants exposed to moonlight flowered 2–3 days late. They suggested that, while full moonlight may be perceived in the photoperiodic reaction, in the natural environment it would only have a very slight effect on the time of flower induction at the most [13]. These studies showed that plants can perceive even very low moonlight but they provided no information about how moonlight is perceived at the molecular level and can affect plant physiology, particularly transcriptional activation. But maybe the findings of these studies need to be reinterpreted in the light of the recent work of P. Barlow [14].

Plants use the circadian clock to synchronize their physiology and development with daily and yearly changes in the environment [15]. The aim of the present study was to investigate whether coffee photoreceptors can perceive moonlight and deregulate circadian clock mechanisms. One key aspect of clock-driven physiological patterns in plants is that they match environmental patterns while relying on accurate prediction of day and night lengths. Genes orthologous to circadian light perception in *Arabidopsis* and genes involved in photosynthesis pathways and regulation are present in the coffee genome. The expression pattern of core clock genes in coffee trees is similar to that in *Arabidopsis*, suggesting a high level of conservation. While studying the circadian cycle of young *Arabica* coffee seedlings in an artificial environment (phytotron, 12/12 h photoperiod), we decided to also check our results by analyzing older plants in the greenhouse. We conducted sampling at three hourly intervals at spring solstice (12 h day, 12 h night). When we analyzed the key core clock gene *LHY* using qRT-PCR, we observed a surprising phenomenon. The gene expression profile showed a completely unexpected peak in the middle of the night. By chance, on the night of our study, the moon was full “at the exquisite hour when a vast and tender peacefulness seems to descend from the firmament” (freely adapted from a poem by Paul Verlaine entitled “The good song”, 1871). To further investigate this discovery, we analyzed our samples using RNAseq and confirmed our results at spring solstice in plants grown under the same environmental conditions, but also in plants grown in a phytotron.

## Results

### Particularities of moonlight

**Fig. 1:**
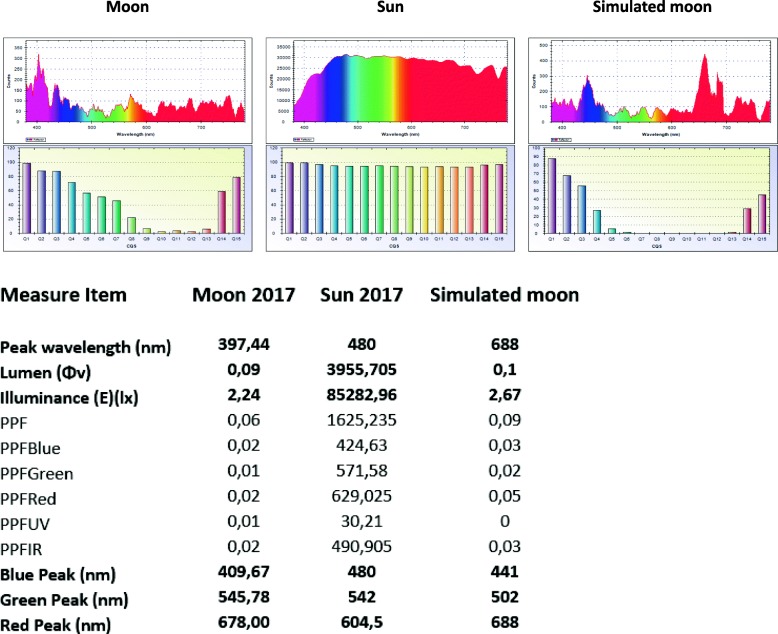
Spectrometer natural and simulated full moonlight and natural sunlight measurements

Different communities worldwide traditionally use lunar rhythms as a tool to pinpoint the best germination and harvest times. The moon can act in two ways on plants, via its gravitational effect or via the sunlight it reflects. The gravitational effect is now well known, but the effect of full moon (FM) light is completely unknown. Compared with sunlight, the wavelength of full moonlight is generally centered around 400 nm (580 nm for the sun) with a very low energy level (0.2 lx or 0.0024 μmol m^− 2^ s^− 1^). The red:far red (R:FR) ratio of sunlight during the day is more than 1.2, while that of moonlight is between 0.18 and 0.22 (Fig. 1).

### Massive transcriptional up and down regulation induced by full moonlight

**Fig. 2:**
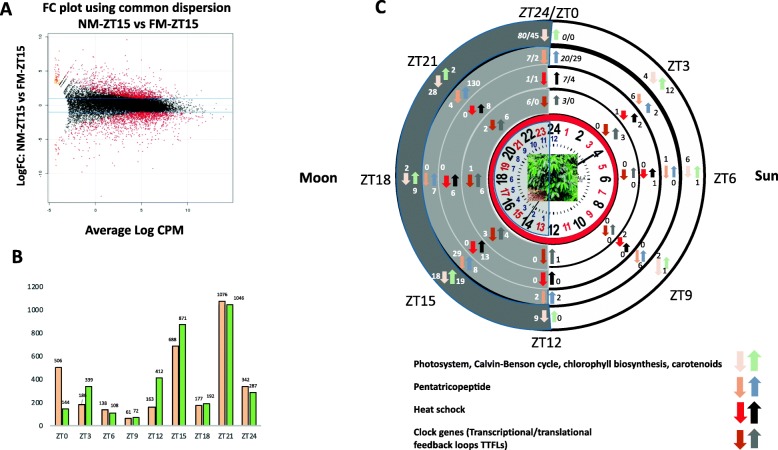
How the full moon clock and the new moon clock differ. **a** Normalization of the data, example for ZT 15: scatterplot of the log fold-change for the FullMoon vs NewMoon comparison against the log-counts-per-million logs in reads for each gene. The log fold-change of the data was centered on 0, showing that the libraries were correctly normalized. In the figure, differentially expressed genes are shown in red (*p* < 0.05) between the two conditions. **b** In response to the FM, many coffee leaf genes were transcriptionally down or up regulated compared with the the response to the NM at the different Zeitgeber times – (ZT0 = dawn, ZT12 = dusk), (color pink = down; color green = up). **c** Examples of these responses (from inside to outside) include histone gene expression, heat shock genes, pentatricopeptide family genes, photosynthesis related genes (photosystem, Calvin cycle, chlorophyll metabolism, carotenoid). Numbers associated with the up or down arrows indicate the number of genes up or down-regulated, respectively, at each ZT. We provide both numbers for ZT24 and ZT0 (ZT24 in italics) (coffee plant photo credit, Breitler jean-christophe)

Full moonlight was reported to induce transcriptional up or down regulation of many coffee leaf genes compared with new moon (NM) light (Fig. 2a). Under our experimental conditions, we monitored transcript accumulation by RNAseq every 3 h over a 24 h period in March 2016 (Additional file 1: Tables 0, 1, 2, 3, 4, 5, 6, 7, 8 and 9). Taking the whole transcriptome (25,574 genes) into account, we observed only slight differences between the FM and NM at ZT6, ZT9, ZT18, with only 0.3 to 1.2% of genes being differentially regulated. We observed two maxima at ZT15 (4.8%) that corresponded to the FM zenith and 3 h later at ZT21, with more than 6.8% of the genes being differentially regulated (Fig. 2b-c). Overall, we found that 3387 genes were differentially regulated. These results clearly demonstrate that moonlight was perceived by the coffee leaves.

### Effect of FM on photoreceptor transcription

**Fig. 3:**
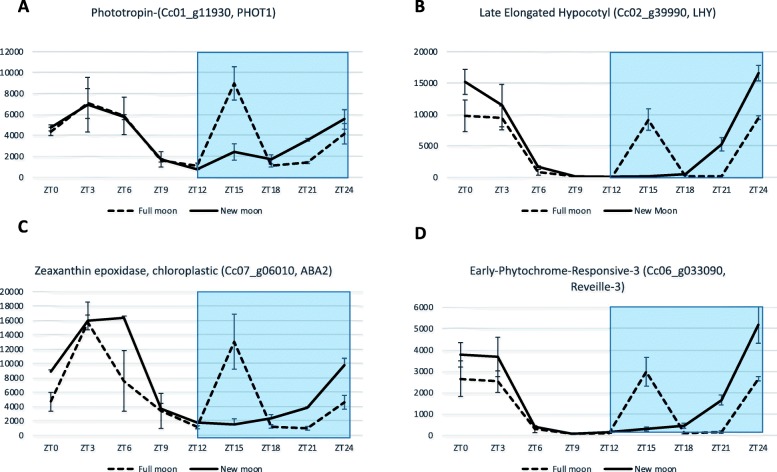
**a** RNAseq expression profile of *PHOT1*, *zeaxanthin epoxidase*, **b** *LHY* and *Reveille 3*. Both genes showed a transcription peak at ZT15, 3 h after the moon zenith. **c** *Zeaxanthin epoxidase* had an expression pattern similar to that of *PHOT1*, **d** while the pattern obtained for *Reveille 3* was similar to that of LHY. RNAseq data were standardized by DESeq2. Full moon (solid line); new moon (dotted); grey: subjective night

Phytochromes (PHY), cryptochromes (CRY), ZEITLUPE (ZTL) family proteins and phototropins (PHOT) are known to be major red/far-red and blue light photoreceptors. It is likely that several of these photoreceptors could be involved in moonlight perception, but most them are unaffected at the transcription level. Only phototropins were highly expressed at the FM zenith (ZT15) (Fig. 3). We observed that *PHOT1* gene expression was highly correlated with several genes involved in chlorophyll biosynthesis. For example, the correlation with the magnesium chelatase gene was r = 0.91 (Fig. 3). Not surprisingly, the *PHOT2* gene, which is known to react to strong blue light, was less differentially expressed than PHOT1 (log2FoldChange 0.69 and 1.40, respectively). Zeaxanthin epoxidase (*ZEP*), beta-carotene 3-hydroxylase (*CRTZ*) and phytoene synthase (*PSY1*) gene expressions were also highly correlated with *PHOT1*. We observed higher gene expression at ZT15, indicating that the carotenoid biosynthesis pathway was activated by full moonlight.

### Key core clock genes are affected by full moonlight

The accumulation of coffee putative clock gene transcripts (LHY, Gigantea, Elf3, Elf4, Lux, PRR 5/7/9, PIF1, PIF4, Constans-like 2/4/9/16) was affected by full moonlight at ZT3, ZT12, ZT15, ZT18, ZT21, ZT24 (Additional file 1: Table 2, 5, 6, 7, 8, 9). In a parallel study using *Arabica* plantlets and RNA sequencing time-course data, we determined the cycling transcripts by running JTK_CYCLE for two cycles (48 h). Out of the 25,574 genes of the whole transcriptome, we found 4126 (16%) rhythmic genes at their level of transcription, including 83% that were similar to *Arabidopsis* rhythmic genes (Additional file 1: Figure S10). Of the 3387 genes differentially expressed between FM and NM, 40% were rhythmic, which is a significantly larger proportion than 18% of the total number of genes (*p* < 0.0001), thus showing that the core clock alteration caused by the FM influenced many genes, with most of them being rhythmic genes.

We found that the accumulation of coffee putative clock gene transcripts (LATE ELONGATED HYPOCOTYL (LHY), TIMING OF CAB EXPRESSION 1 (TOC1), GIGANTEA (GI), EARLY FLOWERING 3 and 4 (Elf3, Elf4), LUX ARHYTHMO (LUX), PSEUDO-RESPONSE REGULATOR (PRR 5, 7, and 9), PHYTOCHROME INTEGRATING FACTOR (PIF1, PIF3, PIF4, PIF7), CONSTANTS-like 2, 4, 9, and 16 (CO)) were affected by full moonlight. Pairwise phase plots (Additional file 1: Figure S11) showed similar relationships between FM and NM, but with unusual full-moon loops, thus illustrating the influence of the FM while changing the relationships between key circadian rhythm genes in a very punctual but marked manner. Taken together, our data suggest that core clock genes are altered in amplitude by the FM (Fig. 2c and Additional file 1: Tables 0, 1, 2, 3, 4, 5, 6, 7, 8, 9 and 10 and Fig. S11). However, the FM also changed the phase of several rhythmic genes (Additional file 1: Figure S12) and led to phase delays (at least 6 h in our study).

### Full moonlight affects the expression of many regulator genes

More than 490 putative pentatricopeptides (PPR) have been predicted in the coffee genome (http://coffee-genome.org/advanced). Here we showed (Fig. 2c) that 130 genes of this family were upregulated at ZT21 while only four were down-regulated. Of the 130 up-regulated *PPR* genes, 97 were rythmics and 127 were negatively correlated with *LHY* gene expression (r ranging from 0.5 to 0.88, *P* < 0.01). At ZT15, 29 *PPR* genes were up-regulated and 8 were down-regulated. We also observed high disequilibrium in ribosomal activity at ZT21, where 69 ribosomal genes were up-regulated and only 4 were down-regulated (data not shown).

### Transcription of photosynthesis-related genes, heat shock and lipid biosynthesis genes is drastically affected by full moonlight

Regarding photosynthesis-related genes, we observed (Fig. 2c), that 50 genes of this pathway were strongly up or down-regulated during the night. Light-harvesting a-b binding proteins (CAB 1C-4/8/21/36) were highly up-regulated at FM at ZT15 and ZT18. On the other hand, a many photosynthesis-related genes were found to be down-regulated before dawn at ZT21, but mostly at ZT24 (Fig. 2c). Logically, several photosynthesis-related genes were highly correlated with major redox genes ((Additional file 1: Table S13) for which they are the main target of redox regulation. Indeed, we observed (Fig. 2c) up-regulation of genes belonging to the heat shock protein family HSFs). Thirteen genes were up-regulated at ZT15, six at ZT18, eight at ZT21, and seven at ZT24, while only one gene was down-regulated at ZT24. The majority of these genes were classified as rhythmic. Many genes of the lipid biosynthesis pathway peaked differentially at ZT15 (Additional file 1: Table S14), showing that the lipid biosynthesis pathway was also altered by full moonlight.

### Coffee trees perceive the moonlight that deregulates their gene expression

**Fig. 4:**
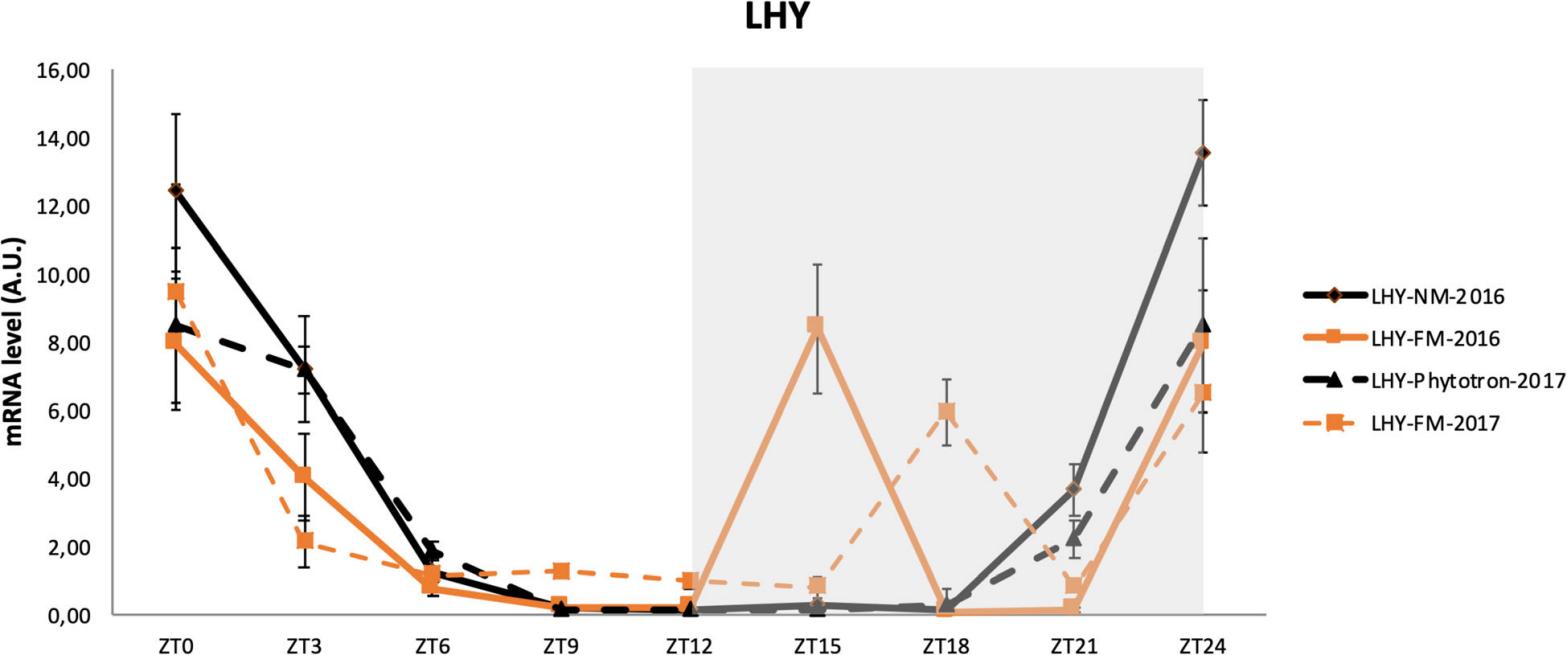
Atypical accumulation of *LHY* transcripts under moonlight exposure. March 2016 NM (solid dark line), March 2016 FM (solid orange line), March 2017 phytotron (dashed dark line) and March 2017 FM (dashed orange line)

At spring solstice in 2016, using RT-QPCR, we assessed the expression of clock genes (LHY, GI, LUX ARRYTHMO, TOC1), chlorophyll biosynthesis genes (Protochlorophyllide Oxidoreductases a (POR1A)), and starch metabolism gene (Alpha-glucan water dikinase 1 (GWD1)), during the FM and NM. We repeated the experiment during the FM in March 2017, with the same plants in the same greenhouse. In this new experiment, we also placed half of the plants in a phytotron (12/12 h photoperiod) where the plants did not receive any light at night throughout the month of March. We found no difference in gene expression between plants exposed to the NM in 2016 and 2017 and plants placed in the phytotron (Additional file 1: Figure S16). This lack of difference is illustrated in Fig. 4 for *LHY*. In addition, these curves can be compared to that showing the *LHY* expression pattern in Fig. 3b obtained with RNASeq data during the NM.

When RT-QPCR was used to compare the expression of LHY, GI, LUX ARRYTHMO, POR1A, POR1B, GWD1 and ISA3 genes between the FM of 2016, 2017 and the NM of 2016 or 2017, we observed the same unexpected peak when the plants were exposed to full moonlight (Fig. 4, Additional file 1: Figure S17). The expression very clearly peaked in 2016, quite similar to the peak observed by RNA-seq (Fig. 3) for all genes under study. However, in 2017 the variations displayed a lower amplitude and the atypical expression peak of *LHY* had shifted to ZT18 and was of lower amplitude than in 2016. The difference between the 2 years was likely due to the partial cloud cover that prevailed during the nights preceding the FM in March 2017.

### Artificial full moonlight deregulates gene expression

**Fig. 5:**
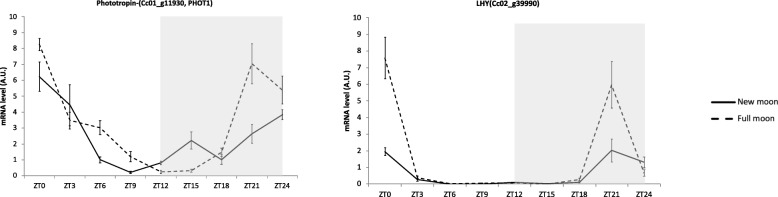
Spectrometer measurements on a NM day, a full sun day and in a growth chamber. We simulated the luminous intensity of the moon in a growth chamber using four types of LED programmed at the main wavelengths 450 nm (blue), 660 nm (red), 730 nm (red) and in white light to emit a light intensity of 6 lx (< 2 PAR). Light intensity spectra (cd) and histograms of the color quality scale (CQS) and light values measured by a Rainbow-Light Portable Spectrometer MR-16 PPF under a full moon, a full sun day and in a growth chamber are shown. *LHY* and *PHOT1* gene expression were analyzed by RT-qPCR. Plants exposed to this artificial ‘moonlight’ showed atypical transcription at ZT21 (dashed line)

In order to confirm the huge impact of weak light on gene transcription, we designed a combination of LEDs to reproduce full moonlight in a growth chamber. We set up four different types of LED lighting to reproduce the bright spectrum of the FM as well as possible (Fig. 1). The ratio between the blue light intensity and green light intensity at the FM zenith was about 1.30, which is quite similar to the 1.41 ratio reproduced in our growth chamber. We regulated the overall intensity at less than 6 lx (0.073 μmol m^− 2^ s^− 1^), so the quantity of energy perceived by the plant was less than 1 photosynthetically active radiation unit (PAR). Technically, we were unable to increase the light intensity to mimic that emitted at moonrise and at the moon zenith. We switched on the light at full intensity at 10:00 pm. Despite the difficulty in reproducing the light of the FM, after 7 days of treatment, plants exposed to this artificial ‘moonlight’ showed atypical transcription at ZT21 of *LHY*, *PHOT1* and *PHOT2* genes in the RT-qPCR analysis (Fig. 5). The night peak was produced at ZT21 instead of ZT15 under natural FM conditions.

## Discussion

Plants are exposed to repeated changes in light quantity and quality and they use a set of photoreceptors to recognize the surrounding light environments [16, 17]. Are these photoreceptors able to perceive full moonlight? The full moonlight PAR is clearly inadequate for photosynthetically supported growth, but from a qualitative viewpoint moonlight mainly consists of blue and far-red light, i.e. two wavelengths perceived by plants and known to affect both their physiology and development [18]. On the other hand, full moonlight can be perceived by plant photoreceptors as it mainly consists of blue light with a very low R:FR ratio. We are almost certain that this is only a moonlight effect, but we cannot completely rule out gravity effects. Plants placed in the phytotron during the FM of 2017 showed the same gene expression profiles as those obtained for the NM of 2017 and 2016. This control shows that it is indeed moonlight that is perceived and not a variation of gravity between the beginning and middle of the month. As the qRT-PCR results were similar for the NM in 2016 and in the culture chamber during the FM of 2017, we could conclude that moonlight was responsible for the gene expression modification, not gravitiational forces.

Phytochromes (PHY), cryptochromes (CRY), ZEITLUPE (ZTL) family proteins and phototropins (PHOT) are known to be major red/far-red and blue light photoreceptors [19, 20]. The PHOT protein acts as a blue light photoreceptor [21]. Zeaxanthin epoxidase (ZEP) is known to respond to red light [22]. It is likely that several of these photoreceptors are involved in moonlight perception. At the transcription level, most of them were unaffected, except phototropins, which were highly expressed at the FM zenith. Phototropins are blue-light receptors controlling a range of responses that serve to optimize the photosynthetic efficiency of plants. These include phototropism, light-induced stomatal opening, and chloroplast movements in response to changes in light intensity [23]. We observed that *PHOT1* gene expression was highly correlated with several genes involved in chlorophyll biosynthesis or within the chloroplast, and also with some genes involved in the carotenoid biosynthesis pathway. Over-expression of ZEP, which is known to respond to red light [22], CRTZ and PSY1 indicated that the carotenoid biosynthesis pathway was activated by full moonlight.

The circadian clock produces rhythmic variations in a suite of biochemical and physiological processes that help to optimize plant growth in daily cycles. Regular environmental changes, especially the sunrise and sunset, coordinate these rhythmic behaviours. Photoreceptors and metabolites produced during photosynthesis, operate to synchronize the internal timing clock with lighting cues. In our study, we hypothesized that massive transcriptional activation would be a good way to demonstrate the effect of moonlight on plants. Moreover, transcript abundance is useful to assess the effects of external clues on circadian oscillations. Light-regulated changes in the morphology of a dicot or monocot seedling are accompanied by an alteration in the expression of up to 20% genes in *Arabidopsis* and rice [24]. The circadian clock provides a mechanism for plants to anticipate events such as sunrise and to adjust their transcriptional programs to coordinate environmental signals and endogenous pathways. Clock activity can be reset by environmental cues such as temperature, photoperiod and metabolic state [25]. A change in ambient light signals induces changes in a molecular pacemaker called the circadian clock [15], which is a biological network of interconnected feedback loops [26]. Here we demonstrated that weak full moonlight had a profound impact on numerous genes, particularly at FM zenith and 3 h later. The main core clock genes were deregulated among the 3387 affected genes.

We observed atypical expression of the main core clock genes during FM when the findings were correlated with those of many other genes like *REVEILLE3* (*REV3*). Several genes showed expression patterns identical to those of core clock genes. *REV3* expression was correlated with *LHY* (*r* = 0.98), suggesting that these two genes were probably co-regulated (Fig. 2). *REV3* plays a photoperiod role in growth regulation [27]. In fact, many genes with patterns similar to *LHY* behave as if a day phase takes place at night. Of the 3387 genes differentially expressed between FM and NM, 40% were rhythmic, indicating that the core clock alteration caused by the FM exerted impacted a large number of genes, including a majority of rhythmic genes.

Among the 3387 deregulated genes, we also observed many genes involved in transcriptional and post-transcriptional processes including ribosomal genes and PRR proteins, respectively. PPR proteins are RNA binding proteins involved in post-transcriptional processes (RNA processing and translation) in mitochondria and chloroplasts, where they can affect gene expression in various ways [28]. Here we hypothesize that, once a plant has perceived moonlight, ribosomal genes and PPR proteins serve as regulatory factors and reprogram nuclear and organellar gene expression earlier.

Regarding photosynthesis-related genes, 50 genes of this pathway were deregulated by moonlight. Many of these genes were found to be down-regulated just before and at dawn, suggesting that full moonlight has a negative effect on the primary photosynthetic machinery at dawn.

We demonstrated that the weak intensity of the FM was able to alter the transcription of many important genes. However, it is still unclear how this transcription alteration is translated phenotypically. Components of the photosynthetic apparatus vary over the course of the day to maximize energy absorption while limiting damage caused by excessive light harvesting. Lai and co-workers showed that the circadian clock coordinates *ROS* homeostasis and the transcriptional response [29]. Here we found that several redox genes which regulate the photosynthetic machinery were remarkably highly correlated with *LHY* (Additional file 1: Table S13). The modification of the transcription of major rhythmic redox genes, many heat shock proteins and carotenoids genes seemed to be prove that the moonlight was perceived as stress by the plant. Activation of stress-responsive pathways is energetically demanding, which raises the question as to what is the plant protecting itself against.

## Conclusion

Could moonlight be an environmental cue perceived by the plant to channel some of its plant resources towards reproduction or defense? These early results pave the way for future studies on the impact of moonlight on plant physiology. FM nights in natural conditions are not easy to study because the sunrise and moonset times vary and weather conditions are not always favorable. Moreover the moon’s trajectory resembles a complex ballet around the earth. Artificial culture conditions can facilitate studies on the effect of moonlight on model plants but also the effects of light pollution on plants. We think that the start of the artificial lunar light was too late in our experiment, which shifted the expression of the genes concerned. However, in artificial conditions, our results confirmed that very low luminous intensities could be perceived by plants and that they had the capacity to modify the transcription of one photoreceptor and one core clock gene.

## Methods

### Plant material and growth conditions

The *C. arabica* var. Caturra seeds came from the La Cumplida Research Center (Matagalpa, Nicaragua). To determine the effects of moonlight, the plants were cultivated in a glasshouse under natural daylight (65–75% humidity, 25 °C temperature, 12/12 h photoperiod) at IRD (Montpellier, France) in 3 L pots containing a GO M2 (Jiffygroup) potting soil mixture with watering as necessary. Leaf samples were collected from 1 year old plants for RNA extraction at Zeitgeber time (ZT) point ZT0 (sunrise), ZT3, ZT6, ZT9, ZT12 (sunset), ZT15, ZT18, ZT21, and ZT24 in March 2016, and from the same plants in March 2017. Sampling was done at the spring solstice FM and the following NM (4 biological replicates). During the FM, samples were also taken from control plants cultivated in a phytotron under artificial light (CRYONEXT, model RTH 1200 L, with the following parameters: 12/12 h light/dark photoperiod, 80% humidity, 25 °C temperature and 600 mmol m^− 2^ s^− 1^ luminosity.

We performed an experiment using the same phytotron and conditions to identify the set of genes with rhythmic expression. We generated a 48 h transcriptomic time-course dataset. Leaves were snap frozen in liquid nitrogen and stored at − 80 °C until RNA analysis. During sampling, three biological replicates were performed using three plants for all RNAseq experiments and four biological replicates with the plants exposed to simulated moonlight. To classify the time points at which the sampling was carried out, we used Zeitgeber time (ZT), which is defined as the time in hours from the start of a normal 12/12 h photoperiod cycle (photoperiod 12 h/12 h). For this purpose, we collected leaf samples at 3 h resolution from ZT0 to ZT24.

### Light analysis

Solar and lunar light was analyzed in 2016 and 2017 at spring solstice using an MR-16v4 *Rainbow*-*Light Portable* Light *Measuring* Instrument. This spectrometer uses micro-electromechanical systems (MEMS) and dynamic thermal equilibrium (DTE) technologies, with high accuracy (spectral deviation in +/− 0.1 nm, measuring difference < 0.3%) and high stability (repeated measurement error < 0.04).

### Simulation of moonlight in a growth chamber using LEDs

In order to simulate the luminous intensity of the moon in a growth chamber, we measured the real luminous intensity emitted by the moon under a FM and NM. We then programmed four types of LED in the growth chamber to emit a light intensity of 6 lx at the main wavelengths: 450 nm (blue), 660 nm (red), 730 nm (red) and in white light. We measured the light intensities to obtain the real intensity value in the growth chamber. We used three devices: a Rainbow-Light Portable Spectrometer (version MR-16 PPF) to generate a light intensity spectrum, a TopSafe light meter to obtain illuminance (lx) and a photometric PAR probe to obtain the photosynthetic photon flux density (PPFD) expressed in μmol/m^2^/s. No background noise was detectable with the light meter or the photometric PAR probe, but the spectrometer showed a background noise spectrum (Fig. 1). The experiment was conducted in a growth chamber at 25 °C and 60% humidity. We placed the LEDs on a shelf and programmed them to emit a light intensity of 350 PAR between ZT0 and ZT12, corresponding to NM conditions. We programmed LEDs on another shelf to emit a light intensity of 350 PAR between ZT0 and ZT12 and of 6 lx between ZT15 and ZT20, corresponding to FM conditions. We exposed 10 *Coffea arabica* plants to NM conditions for 10 days to acclimatize them to the growth chamber. We then exposed 5 plants among the 10 acclimated plants in the FM conditions for 7 days. At the end of the 7 day period, the first sample was taken at ZT0, and then every 3 h for 24 h. Samples (4 biological replicates) were taken from 5 plants per condition. The samples were taken from the 3rd and 4th leaves of the coffee plants.

### RNA isolation

Total RNA was extracted from leaves prefrozen in liquid nitrogen that were subsequently ground and processed as described previously [30]. RNA quantification was performed using a NanoDropTM 1000 Spectrophotometer (Thermo Fisher Scientific, Waltham, MA, USA) and the quality was assessed using the Agilent 2100 Bioanalyzer system with the RNA 6000 Nano™ kit.

### Real-time RT-qPCR assays

PCR experiments were performed as previously described [31]. Primers were designed using Primer3Plus web-based software (http://www.bioinformatics.nl/cgi-bin/primer3plus/primer3plus.cgi). Based on published data, we targeted three key genes of the circadian clock *CcLHY* (Cc02_g39990), *CcGIGANTEA* (Cc10_g15270) and *CcLUX-ARRYTHMO* (Cc06_g20160). The specificity of the PCR products generated for each primer set was checked by analyzing the Tm (dissociation) of the amplified products. PCR efficiency (*E*) was estimated using absolute fluorescence data captured during the exponential phase of amplification of each reaction with the eq. (1 + *E*) = 10^(− 1/slope)^ (Ramakers et al. 2003) (Additional file 1: Table S15). Expression levels were calculated by applying the formula (1 + *E*)^−ΔΔ*C*t^, where ^Δ*C*^t,target^=*C*^t,targetgene^−*C*^t,*CaGAPDH* ^and ΔΔ*C*^t^=Δ*C*^t,target^−Δ*C*^t, reference sample, with the *T*_0_ sample used as reference for each construct. Expression levels were normalized with the expression of the *CaGAPDH* gene (GB accession number GW445811 using primer pair GAPDH-F/R) serving as endogenous control [32].

### RNA sequencing and bioinformatics analysis

RNA sequencing (RNAseq) was carried out by the MGX platform (Montpellier GenomiX, *Institut de Génomique Fonctionnelle*, Montpellier, France; www.mgx.cnrs.fr/). RNAseq libraries were constructed with the TruSeq Stranded mRNA Sample Preparation kit from Illumina. One microgram of total RNA was used for the library construction. SuperScript IV reverse transcriptase and random primers were used to produce first strand cDNA from cleaved RNA fragments. This was followed by second-strand cDNA synthesis. The cDNA fragments were repaired, before the addition of a single ‘A’ base and the subsequent ligature of the adapter. The final cDNA libraries were validated with a Bioanalyzer kit (Standard Sensitivity NGS) and quantified by qPCR (ROCHE Light Cycler 480). Libraries were pooled in equal proportions, before denaturation with NaOH and dilution to 17 pM, and before clustering on two lanes in a flow cell. Clustering and 100 nt single read sequencing were performed with a Hiseq 2500 according to the manufacturer’s instructions. Image analysis and base calling were performed using HiSeq Control Software (HCS) and the Real-Time Analysis component (Illumina). The data quality was assessed using FastQC from the Babraham Institute (http://www.bioinformatics.babraham.ac.uk/projects/fastqc/) and Illumina Sequence Analysis Viewer (SAV) software. We obtained an average of 21 million single end reads per sample.

### Differential expression analysis

Before differential expression (DE) analysis, genes whose sum of counts (by summing the counts per repetition (3)) was below 45 were discarded. Reads were then standardized across libraries using the normalization procedure in DESeq2 [33]. FM/NM comparisons were performed at ZT0, ZT3, ZT6, ZT9, ZT12, ZT15, ZT18, ZT21 and ZT24. Differential expression was considered statistically significant at *p* < 0.05. All genes of interest were analyzed and compared using the TopHat2 2.1.1 (with Bowtie 2.2.9) algorithm against the *Coffea canephora* genome (Coffee Genome Hub) (splice junction mapping) and BWA-backtrack 0.7.15 algorithm against the *Coffea arabica* transcriptome [34] (mapping and filtering).

### Statistics

Differential expression (DE) analysis was performed using R 3.4.2 software and the DESeq2 1.18.1 package. Rhythmic gene expression, period and phase parameters were measured using JTK_CYCLE implemented in MetaCycle v1.1.0 [35].. To identify the rhythmic transcripts, we analyzed the DESeq2 normalized data. JTK_CYCLE uses a non-parametric test to detect cycling transcripts [36]^.^ We considered transcripts with Benjamini-Hochberg q values (BH.Q) < 0.05 as rhythmic transcripts. JTK-CYCLE was run with a 21–27 h range of periods. A χ^2^ test *(P < 0.05)* was used to determine if the rhythmic genes in the differential expressed gene set were present in greater numbers than expected by chance. Graphs were plotted using Excel, or R. The R codes are available from the corresponding author.

## Supplementary information

**Additional file 1: Table S0.** REPLICATES. **Table S1.** Genes differentially expressed between Full moon and new Moon at ZT0. **Table S2.** Genes differentially expressed between Full moon and new Moon at ZT3. **Table S3.** Genes differentially expressed between Full moon and new Moon at ZT6. **Table S4.** Genes differentially expressed between Full moon and new Moon at ZT9. **Table S5.** Genes differentially expressed between Full moon and new Moon at ZT12. **Table S6.** Genes differentially expressed between Full moon and new Moon at ZT15. **Table S7.** Genes differentially expressed between Full moon and new Moon at ZT18. **Table S8.** Genes differentially expressed between Full moon and new Moon at ZT21. **Table S9.** Genes differentially expressed between Full moon and new Moon at ZT24. **Figure S10.** Rhythmic genes in common between Coffee and *Arabidopsis thaliana*. **Figure S11.** Pairwise phase plots of the core clock genes. **Figure S12.** Number of genes per phase under new moon and the full moon. **Table S13.** Correlation matrix between Redox homeostasis genes and LHY. **Table S14.** Correlation matrix between genes involved in lipids pathways and LHY. **Table S14.** Primers used for genes studied by qRT-PCR. **Figure S16.** RT-QPCR of LHY gene during new moon. **Figure S17.** RT-QPCR time-course of core clock genes, POR1A and GWD1.

## Data Availability

All data generated or analysed during this study are included in this published article and its additional files.

## Abbreviations

DTE: Dynamic thermal equilibrium
FM: Full moon
HSP: Heat shock protein
NM: New moon
PAR: Photosynthetically active radiation unit
PPFD: Photosynthetic photon flux density
PPR: Putative pentatricopeptides
ZT: Zeitgeber time

## References

[1] Raven J, Cockell C (2006). Influence on photosynthesis of starlight, moonlight, Planetlight, and light pollution (reflections on Photosynthetically active radiation in the universe). Astrobiology.

[2] Kronfeld-Schor N, Dominoni N, de la Iglesia H, Levy O, Herzog E, Dayan T, Helfrich-Forster C (2013). Chronobiology by moonlight. Proc. R. Soc. B.

[3] Chaffey N, Volkmannb D, Baluškab F (2019). The botanical multiverse of Peter Barlow. Commun Integ Biol.

[4] Gallep C, Viana J, Cifra M, Clarke D, Robert D (2018). Peter Barlow’s insights and contributions to the study of tidal gravity variations and ultra-weak light emissions in plants. Ann Botany.

[5] Beeson C (1946). The moon and plant growth. Nature.

[6] Kolisko L (1936). The moon and the growth of plants. Published by bray-on-Thames.

[7] Semmens E (1947). Chemical effects of moonlight. Nature.

[8] Darwin C. The Power of Movement in Plants. London: J. Murrey, 2nd, p 394 (1880).

[9] Levy O, Appelbaum L, Leggat W, Gothlif Y, Hayward DC, Miller DJ, Hoegh-Guldberg O (2007). Light- responsive cryptochromes from a simple multicellular animal, the coral Acropora millepora. Science.

[10] Bünning E, Mose I (1969). Interference of moonlight with the photoperiodic measurement of time by plants and their adaptive reaction. Proc Natl Acad Sci.

[11] Ffrench-Constant R, Somers-Yeates R, Bennie J, Economou T, Hodgson D, Spalding A, McGregor P (2016). Light pollution is associated with earlier tree budburst across the United Kingdom. Proc. R. Soc.

[12] Gaston K, Bennie J, Davies T, Hopkins J (2013). The ecological impacts of nighttime light pollution: a mechanistic appraisal. Biol Rev.

[13] Kadman-Zahavi A, Peiper D (1987). Effects of moonlight on flower induction in *Pharbitis nil*, using a single dark period. Ann Bot.

[14] Barlow P (2015). Leaf movements and their relationship with the lunisolar gravitational force. Ann Bot.

[15] Mora-Garcia S, de Leone M, Yanovsky M (2017). Time to grow: circadian regulation of growth and metabolism in photosynthetic organisms. Curr Opin Plant Biol.

[16] Kozuka T, Suetsugu N, Wada M, Nagatani A (2012). Antagonistic regulation of leaf flattening by Phytochrome B and Phototropin in Arabidopsis thaliana. Plant Cell Physiol.

[17] Seluzicki A, Burko Y, Chory J (2017). Dancing in the dark: darkness as a signal in plants. Plant, Cell & Environ.

[18] De Wit M, Costa Galvao V, Fankhauser C (2016). Light-mediated hormonal regulation of plant growth and development. Annu Rev Plant Biol.

[19] Costa Galvao V, Fankhauser C (2015). Sensing the light environment in plants: photoreceptors and early signaling steps. Curr Opin Neurobiol.

[20] Kong S-G, Okajima K (2016). Diverse photoreceptors and light responses in plants. J Plant Res.

[21] Briggs W, Christie J (2002). Phototropins 1 and 2: versatile plant blue-light receptors. Tr Pl Sci.

[22] Oh E, Yamaguchi S, Hu J, Yusuke J, Jung B, Paik I, Lee H-S, Sun T-P, Kamiya Y, Choi G (2007). PIL5, a Phytochrome-interacting bHLH protein, regulates gibberellin responsiveness by binding directly to the GAI and RGA promoters in Arabidopsis seeds. Plant Cell.

[23] Christie J (2007). Phototropin blue-light receptors. Annu Rev Plant Biol.

[24] Jiao Y, Ma L, Strickland E, Deng X (2005). Conservation and divergence of light-regulated genome expression patterns during seedling development in Rice and Arabidopsis. Plant Cell.

[25] Millar A (2016). The intracellular dynamics of circadian clocks reach for the light of ecology and evolution. Annu Rev Plant Biol.

[26] Hsu Y, Harmer S (2014). Wheels within wheels: the plant circadian system. Tr Pl Sci..

[27] Gray J, Shalit-Kaneh A, Chu D, Hsu P, Harmer S (2017). The REVEILLE clock genes inhibit growth of juvenile and adult plants by control of cell size. Plant Physiol.

[28] Barkan A, Small I (2014). Pentatricopeptide repeat proteins in plants. Annu Rev Plant Biol.

[29] Lai A, Doherty C, Mueller-Roeber B, Kay S, Schippers J, Dijkwel P (2012). Circadian clock-associated 1 regulates ROS homeostasis and oxidative stress responses. Proc Natl Acad Sci.

[30] Breitler J-C, Campa C, Georget F, Bertrand B, Etienne H (2016). A single-step method for RNA isolation from tropical crops in the field. Sci Rep.

[31] Marraccini P, Vinecky F, Alves GS, Ramos H, Elbelt S, Vieira N, Carneiro F, Sujii P, Alekcevetch J, Silva V, DaMatta F, Ferrão M, Leroy T, Pot D, Vieira L, da Silva F, Andrade A (2012). Differentially expressed genes and proteins upon drought acclimation in tolerant and sensitive genotypes of Coffea canephora. J Exp Bot.

[32] Alves GS, Torres LF, Déchamp E, Breitler JC, Joët T, Gatineau F, Andrade A, Bertrand B, Marraccini P, Etienne H (2017). Differential fine-tuning of gene expression regulation in coffee leaves by CcDREB1D promoter haplotypes under water deficit. J Exp Bot.

[33] Love M, Huber W, Anders S (2014). Moderated estimation of fold change and dispersion for RNA-seq data with DESeq2. Genome Biol.

[34] Ivamoto S, Reis Júnior O, Silva Domingues D, dos Santos T, de Oliveira F, Pot D, Leroy T, Esteves Vieira L, Falsarella Carazzolle M, Guimarães Pereira G, Protasio PL (2017). Transcriptome analysis of leaves, Flowers and Fruits Perisperm of *Coffea arabica* L Reveals the Differential Expression of Genes Involved in Raffinose Biosynthesis. PLoS ONE.

[35] Wu G, Anafi R, Hughes M, Kornacker K, Hogenesch J (2016). MetaCycle: an integrated R package to evaluate periodicity in large scale data. Bioinformatics..

[36] Hugues M, Hogenesch J, Kornacker K (2010). JTK_CYCLE: an efficient non-parametric algorithm for detecting rhythmic components in genome-scale datasets. J Biol Rhythm.

